# Níveis altos de marcadores inflamatórios sistêmicos associados à incidência de metástase em osteossarcoma

**DOI:** 10.1055/s-0045-1810033

**Published:** 2025-09-08

**Authors:** I. Gede Eka Wiratnaya, Mohamad Dimas Ismail, Risang Haryo Raditya, I. Putu Dema Prasetya

**Affiliations:** 1 Departamento de Ortopedia e Traumatologia, Faculdade de Medicina, Udayana University, Prof. Ngoerah General Hospital, Bali, Indonésia

**Keywords:** L-lactato desidrogenase, neoplasias ósseas, osteossarcoma, proteína C-reativa, sedimentação sanguínea, blood sedimentation, bone neoplasms, C-reactive protein, L-lactate dehydrogenase, osteosarcoma

## Abstract

**Objetivo:**

Este estudo explora a associação entre esses marcadores inflamatórios e metástase em pacientes com osteossarcoma.

**Métodos:**

Realizamos uma análise retrospectiva de pacientes com osteossarcoma de nosso centro entre janeiro de 2022 e agosto de 2024. Coletamos dados clínicos e laboratoriais dos pacientes, incluindo contagem diferencial de leucócitos, níveis de proteína C reativa (PCR), velocidade de hemossedimentação (VHS), razão neutrófilo-linfócito (RNL) e concentrações de fosfatase alcalina (FA) e lactato desidrogenase (LDH). A correlação estatística entre esses marcadores e metástase foi avaliada por meio dos teste do Qui-quadrado de Pearson, exato de Fisher e regressão logística.

**Resultados:**

O estudo incluiu 40 pacientes com osteossarcoma. Níveis altos de contagem de neutrófilos ( *p* = 0,024; razão de chances [RC] = 12,667; IC95% = 1,402–114,419) de PCR ( *p* < 0,001; RC = 17,000; IC95% = 3,464–83,436), VHS ( *p* = 0,009; RC = 19,000; IC95% = 2,119–170,383), RNL ( *p* = 0,006; RC = 7,429; IC95% = 1,778–31,040) e LDH ( *p* = 0,009; RC = 9,333; IC95% = 2,180–39,962) foram significativamente associados à presença de metástase.

**Conclusão:**

A contagem de neutrófilos, a VHS, a RNL e os níveis de PCR e LDH estão significativamente associados à metástase em osteossarcoma, e podem ser marcadores prognósticos valiosos. Pesquisas futuras devem se concentrar em elucidar os mecanismos subjacentes a essa relação e explorar intervenções terapêuticas direcionadas à inflamação para mitigar a metástase.

## Abstract (en)

**Objective:**

The present study explores the association between these inflammatory markers and metastasis in osteosarcoma patients.

**Methods:**

We conducted a retrospective analysis of osteosarcoma patients at our center between January 2022 and August 2024. We collected the clinical and laboratory data of the patients, including white blood cell differential count, C-reactive protein (CRP) levels, erythrocyte sedimentation rate (ESR), neutrophil-to-lymphocyte ratio (NLR), alkaline phosphatase (ALP) and lactate dehydrogenase (LDH) levels. Statistical correlation between these markers and metastasis was assessed using the Pearson's Chi-squared test, the Fisher's exact test, and logistic regression.

**Results:**

A total of 40 osteosarcoma patients were included. Elevated levels of neutrophil count ( *p* = 0.024; odds ratio [OR] = 12.667; 95%CI = 1.402–114.419), CRP ( *p* < 0.001; OR = 17.000; 95%CI = 3.464–83.436), ESR ( *p* = 0.009; OR = 19.000; 95%CI = 2.119–170.383), NLR ( *p* = 0.006; OR = 7.429; 95%CI = 1.778–31.040), and LDH ( *p* = 0.009; OR = 9.333; 95%CI = 2.180–39.962) were significantly associated with the presence of metastasis.

**Conclusion:**

The neutrophil count, ESR, NLR, and the levels of CRP and LDH are significantly associated with metastasis in osteosarcoma, and they may serve as valuable prognostic markers. Future research should focus on elucidating the mechanisms underlying this relationship and exploring therapeutic interventions targeting inflammation to mitigate metastasis.

## Introdução

O osteossarcoma é o tumor ósseo primário maligno mais comum, e afeta principalmente crianças e adultos jovens. Apesar dos avanços nos protocolos de tratamento que incorporam cirurgia e quimioterapia, o osteossarcoma ainda é associado a uma alta incidência de metástases, em especial nos pulmões, o que contribui significativamente para o prognóstico ruim observado em muitos pacientes. 1 A metástase é a principal causa de mortalidade no osteossarcoma. A taxa de sobrevida em 5 anos é inferior a 30% em pacientes com doença metastática, e de cerca de 70% em pacientes com doença localizada. A compreensão dos mecanismos subjacentes à metástase e a identificação de biomarcadores que predizem o potencial metastático são cruciais para a melhora dos desfechos clínicos. 2

Uma área que tem recebido cada vez mais atenção nos últimos anos é o papel da inflamação na progressão do câncer. A inflamação, como parte da resposta imunológica do corpo, há muito tem sido implicada no início e no desenvolvimento de tumores, e um crescente conjunto de evidências sugere que a inflamação crônica pode facilitar a metástase tumoral. 3 Essa relação é mediada pelo microambiente tumoral, que inclui várias células imunes, citocinas e outros mediadores inflamatórios que interagem com as células tumorais para promover sua proliferação, invasão e migração. 4

Neste contexto, marcadores inflamatórios sistêmicos, como a proteína C reativa (PCR), a velocidade de hemossedimentação (VHS), a razão neutrófilo-linfócito (RNL) e a razão plaqueta-linfócito (RPLR), surgiram como possíveis indicadores do prognóstico do câncer e do potencial metastático em uma ampla gama de tumores malignos. 5 Esses marcadores refletem a resposta inflamatória sistêmica, que, quando desregulada, pode contribuir para a progressão tumoral e o desenvolvimento de metástase. Níveis elevados desses marcadores têm sido associados a desfechos clínicos desfavoráveis, incluindo aumento da agressividade tumoral e disseminação metastática em alguns cânceres, como colorretal, de mama e de pulmão. 6 7 8

No osteossarcoma, o papel dos marcadores inflamatórios como fatores preditivos de metástase é uma área de investigação ativa. Diversos estudos 9 10 11 sugeriram que níveis elevados de marcadores inflamatórios estão associados a piores desfechos de sobrevida, o que sugere que esses marcadores podem ser ferramentas prognósticas úteis na prática clínica. No entanto, os mecanismos precisos pelos quais esses marcadores inflamatórios influenciam a metástase do osteossarcoma não são compreendidos por completo.

A compreensão da relação entre inflamação e metástase no osteossarcoma pode ter implicações clínicas significativas. Primeiramente, a identificação de biomarcadores inflamatórios confiáveis de metástase pode aprimorar a estratificação de risco, o que permite abordagens terapêuticas personalizadas e intervenções mais precoces em pacientes de alto risco. Além disso, o foco nas vias inflamatórias envolvidas na progressão do osteossarcoma pode oferecer novas estratégias terapêuticas para prevenir ou mitigar a metástase, o que pode melhorar os desfechos de sobrevida nessa neoplasia maligna agressiva.

Este estudo teve como objetivo investigar a correlação entre marcadores inflamatórios e a incidência de metástase em pacientes com osteossarcoma. Ao avaliar os níveis de marcadores inflamatórios sistêmicos importantes, como PCR, VHS, RNL e RPL, em pacientes com osteossarcoma, tentamos elucidar seu possível papel como biomarcadores preditivos de doença metastática.

## Materiais e Métodos

### Delineamento Experimental

Este estudo utilizou uma metodologia retrospectiva, que abrangeu pacientes diagnosticados com osteossarcoma com base em achados histopatológicos. A coorte foi composta por indivíduos diagnosticados com osteossarcoma em nossa instituição entre janeiro de 2022 e agosto de 2024. As variáveis independentes analisadas incluíram sexo, idade, localização do tumor e marcadores inflamatórios (PCR, VHS, fosfatase alcalina [FA], lactato desidrogenase [LDH] e RNL), enquanto a variável dependente foi a incidência de metástase. Exploramos como esses vários fatores independentes se relacionam e influenciam a probabilidade de metástase em indivíduos com osteossarcoma.

### População de Pacientes

Os critérios de elegibilidade foram: 1) confirmação histológica do diagnóstico de osteossarcoma por biópsia; 2) avaliação de hemograma completo (incluindo PCR, VHS, RNL, FA e LDH) antes da biópsia cirúrgica; e 3) identificação de metástase em exames radiológicos. Por outro lado, excluímos indivíduos com outras comorbidades, como imunodeficiência, infecções e diabetes mellitus. Além disso, pacientes com malignidades de baixo grau e aqueles sem resultados completos de amostras de sangue para análise também foram excluídos. O comitê de ética de nossa instituição aprovou o estudo, sob o número de protocolo 2024.02.1.0930. Não houve necessidade de obtenção de consentimento livre e esclarecido, uma vez que este estudo não realizou intervenções em pacientes.

### Coleta de Dados

A partir dos prontuários médicos, registramos sistematicamente informações demográficas e clínicas, incluindo sexo, idade, local do tumor, presença de metástase, local da metástase e níveis séricos de PCR, VHS, RNL, FA e LDH. Os resultados do hemograma completo foram obtidos no primeiro atendimento em nossa unidade. Os pacientes foram categorizados com base na metástase. Para mitigar possíveis vieses, estabelecemos critérios claros de inclusão e exclusão para a população do estudo. Além disso, os autores responsáveis pela seleção e análise dos dados dos pacientes não tinham conhecimento dos desfechos do osteossarcoma. Protocolos e instrumentos padronizados foram empregados em nossa instituição para mensurar as variáveis relevantes e, assim, minimizar o viés de informação. Além disso, os dados dos prontuários médicos hospitalares foram corroborados com os laudos de nosso Departamento de Ortopedia e Traumatologia.

### Análise Estatística

A análise de correlação foi feita pelo teste do Qui-quadrado de Pearson e o teste exato de Fisher para a avaliação da associação entre características clínicas e laboratoriais e a incidência de metástase. Os dados clínicos e laboratoriais foram submetidos à análise de regressão logística para determinar quais características continuaram significativas à avaliação concomitante. As variáveis incluídas nessa análise apresentaram valores de *p* < 0,05 no exame de correlação bivariada anterior.

## Resultados

### Dados Demográficos e Características Clínicas

Este estudo contou com 40 pacientes que atenderam aos critérios de inclusão. Todos os pacientes foram diagnosticados com osteossarcoma em nossa instituição entre janeiro de 2022 e agosto de 2024. Usando um método de amostragem total, todos os pacientes elegíveis foram incluídos sem seleção aleatória. Então, os pacientes foram divididos em dois grupos, com ou sem metástase, com base nos resultados do exame radiológico. A amostra foi composta por 20 (50%) homens e 20 (50%) mulheres. A proporção de pacientes na primeira, segunda, terceira e quarta ou mais décadas de vida foi de 3 (7,5%), 21 (52,5%), 5 (12,5%) e 11 (27,5%), respectivamente. A idade mediana foi de 18 (variação: 9–82) anos. As proporções de localização do tumor fora, de 22 (55%) casos no fêmur, 7 (17,5%) casos na tíbia, 7 (17,5%) casos no úmero, e 4 casos em outras localizações: 1 (2,5%) na fíbula, 1 (2,5%) no rádio, 1 (2,5%) na ulna, e 1 (2,5%) na clavícula. Entre os pacientes com metástase, 18 (90%) casos foram detectados no pulmão, e 2 (10%), no cérebro ( Tabela 1 ).

**Tabela 1:** Características clínicas dos pacientes

Variável	Pacientes (n = 40)
**Idade mediana (variação) em anos**	18 (9–82)
1ª década	3 (7,5%)
2ª década	21 (52,5%)
3ª década	5 (12,5%)
≥ 4ª década	11 (27,5%)
**Sexo**	
Masculino	20 (50%)
Feminino	20 (50%)
**Local**	
Fêmur	22 (55%)
Tíbia	7 (17,5%)
Fíbula	1 (2,5%)
Úmero	7 (17,5%)
Rádio	1 (2,5%)
Ulna	1 (2,5%)
Clavícula	1 (2,5%)
**Presença de metástase**	
Sim	20 (50%)
Não	20 (50%)
**Local da metástase**	
Pulmão	18 (90%)
Cérebro	2 (10%)

### Correlação Entre Resultados Clínicos e Laboratoriais e Metástase

Os resultados dos exames de sangue periférico demonstraram que os níveis de neutrófilos, monócitos, FA, LDH, VHS, PCR e RNL no grupo com metástase foram maiores em comparação ao grupo de controle. Os resultados laboratoriais da análise de sangue periférico também demonstraram a distribuição anormal da maioria dos marcadores (neutrófilos, monócitos, eosinófilos, basófilos, FA, LDH, PCR, VHS e RNL), à exceção dos linfócitos. Além disso, os dados com distribuição anormal foram analisados pelo teste de Mann-Whitney, ao passo que os com distribuição normal, pelo teste *t* independente. Os resultados da análise indicaram que neutrófilos ( *p* = 0,01), monócitos ( *p* = 0,033), LDH ( *p* < 0,001), VHS ( *p* < 0,001), PCR ( *p* < 0,001) e RNL ( *p* = 0,012) têm uma correlação estatisticamente significativa com a incidência de metástase em pacientes com osteossarcoma ( Tabela 2 ).

**Tabela 2:** Correlação entre marcador inflamatório e presença de metástase

Variável	Metástase (média ± desvio padrão)	Sem metástase (média ± desvio padrão)	Valor de *p*
**Neutrófilos**	8,10 ± 3,94	5,05 ± 1,66	0,010 ^b^
**Linfócitos**	2,04 ± 0,54	2,04 ± 0,74	0,240 ^a^
**Monócitos**	0,81 ± 0,47	0,52 ± 0,17	0,033 ^b^
**Eosinófilos**	0,21 ± 0,24	0,32 ± 0,26	0,091 ^b^
**Basófilos**	0,03 ± 0,02	0,04 ± 0,02	0,445 ^b^
**Fosfatase alcalina**	382,95 ± 367,71	180,20 ± 107,46	0,076 ^b^
**Lactato desidrogenase**	674,25 ± 828,77	240,35 ± 134,15	< 0,001 ^b^
**Velocidade de hemossedimentação**	67,47 ± 31,95	20,30 ± 1,84	< 0,001 ^b^
**Proteína C- reativa**	53,89 ± 53,63	4,24 ± 5,30	< 0,001 ^b^
**Razão neutrófilo-linfócito**	4,20 ± 2,47	2,49 ± 1,67	0,012 ^b^

**Notas:**^a^ teste *t* independente; ^b^ teste de Mann-Whitney.

A análise bivariada revelou que um alto nível de vários marcadores inflamatórios, como neutrófilos ( *p* = 0,008; razão de chances [RC] = 2,296; IC95% = 1,393–3,784), monócitos ( *p* = 0,035; RC = 2,250; IC95% = 1,562–3,242), VHS ( *p* = 0,001; RC = 7,207; IC95% = 1,092–47,577), PCR ( *p* < 0,001; RC = 4,636; IC95% = 1,610–13,350), RNL ( *p* = 0,004; RC = 2,513; IC95% = 1,283–4,919), FA ( *p* = 0,017; RC = 2,333; IC95% = 1,592–3,421) e LDH ( *p* = 0,001; RC = 3,273; IC95% = 1,329–8,060) foram significativamente associados à incidência de metástase em pacientes com osteossarcoma ( Tabela 3 ).

**Tabela 3:** Análise bivariada de marcadores inflamatórios e incidência de metástases

Variável	Metástase	Sem metástase	Valor de *p*	Razão de chaces (IC95%)
**Neutrófilos**			**0,008**	2,296 (1,393–3,784)
Elevados	8 (88,9%)	1 (11,1%)		
Normais	12 (38,7%)	19 (61,3%)		
**Linfócitos**			−	−
Elevados	0 (0%)	0 (0%)		
Normais	20 (50%)	20 (50%)		
**Monócitos**			**0,035**	2,250 (1,562–3,242)
Elevados	4 (100%)	0 (0%)		
Normais	16 (44,4%)	20 (55,6%)		
**Eosinófilos**			0,376	0,630 (0,194–2,039)
Elevados	2 (33,3%)	4 (66,7%)		
Normais	18 (52,9%)	16 (47,1%)		
**Basófilos**			−	−
Elevados	0 (0%)	0 (0%)		
Normais	20 (50%)	20 (50%)		
**Fosfatase alcalina**			**0,017**	2,333 (1,592–3,421)
Elevada	5 (100%)	0 (0%)		
Normal	15 (42,9%)	20 (57,1%)		
**Lactato desidrogenase**			**0,001**	3,273 (1,329–8,060)
Elevada	16 (72,7%)	6 (27,3%)		
Normal	4 (22,2%)	12 (77,8%)		
**Velocidade de hemossedimentação**			**0,001**	7,207 (1,092–47,577)
Elevada	19 (65,5%)	10 (34,5%)		
Normal	1 (9,1%)	10 (90,9%)		
**Proteína C- reativa**			**< 0,001**	4,636 (1,610–13,350)
Elevada	17 (77,3%)	5 (22,7%)		
Normal	3 (16,7%)	15 (83,3%)		
**Razão neutrófilo-linfócito**			**0,004**	2,513 (1,283–4,919)
Elevada	13 (76,5%)	4 (23,5%)		
Normal	7 (30,4%)	16 (69,6%)		

A análise multivariada mostrou que, entre os marcadores inflamatórios elevados, altos valores de neutrófilos ( *p* = 0,024; RC = 12,667; IC95% = 1,402–114,419), VHS ( *p* = 0,009; RC = 19,000; IC95% = 2,119–170,383), PCR ( *p* < 0,001; RC = 17,000; IC95% = 3,464–83,436), RNL ( *p* = 0,006; RC = 7,429; IC95% = 1,778–31,040) e LDH ( *p* = 0,009; RC = 9,333; IC95% = 2,180–39,962) foram associados à incidência de metástase em pacientes com osteossarcoma ( Tabela 4 ).

**Tabela 4:** Análise multivariada de marcadores inflamatórios e incidência de metástases

Marcadores inflamatórios	Valor	Erro padrão	IC95%	Razão de chances	Valor de *p*
**Neutrófilos**	2,539	1,123	1,402–114,419	12,667	**0,024**
**Lactato desidrogenase**	2,234	0,742	2,180–39,962	9,333	**0,003**
**Velocidade de hemossedimentação**	2,944	1,119	2,119–170,383	19,000	**0,009**
**Proteína C- reativa**	2,833	0,812	3,464–83,436	17,000	**< 0,001**
**Razão neutrófilo-linfócito**	2,005	0,730	1,778–31,040	7,429	**0,006**

## Discussão

Os resultados deste estudo indicam que níveis elevados de neutrófilos, PCR, VHS, RNL e LDH estão significativamente associados à presença de metástase em pacientes com osteossarcoma. Esses resultados corroboram os da literatura existente acerca do papel da inflamação sistêmica na progressão do câncer, em especial no desenvolvimento de metástase.

### Inflamação como Desencadeador de Metástase

A inflamação crônica há muito é reconhecida como uma característica marcante da progressão do câncer. 12 No osteossarcoma, a inflamação no microambiente tumoral cria um ambiente favorável para que as células neoplásicas escapem da vigilância imunológica e promovam a angiogênese, o que facilita a invasão e a metástase. 13 14 A inflamação associada ao tumor não é apenas um efeito colateral: contribui ativamente para a agressividade do câncer por meio de diversas vias. Citocinas pró-inflamatórias, quimiocinas e células imunes são componentes integrais desse processo, pois criam um ambiente que favorece o crescimento, a sobrevida e a disseminação das células tumorais. 15

No osteossarcoma, a metástase, particularmente para os pulmões, é um determinante crítico do prognóstico. O osteossarcoma metastático é caracterizado por um comportamento tumoral mais agressivo, frequentemente associado a um maior grau de inflamação sistêmica. 1 16 Os marcadores identificados neste estudo (PCR, VHS, RNL e LDH) refletem aspectos distintos, mas interconectados, desta resposta inflamatória, e indicam mecanismos pelos quais a inflamação pode impulsionar a progressão metastática.

### Proteína C Reativa (PCR) e Metástase

A PCR é uma proteína de fase aguda sintetizada no fígado em resposta a citocinas pró-inflamatórias, principalmente interleucina 6 (IL-6) e fator de necrose tumoral alpha (FNT-α). 17 Níveis elevados de PCR têm sido consistentemente associados a um prognóstico ruim e a um aumento do potencial metastático em vários tipos de câncer, incluindo o osteossarcoma. 6 18 19 20 A associação significativa entre níveis elevados de PCR e a presença de metástase neste estudo sugere que a PCR pode ser um marcador substituto da extensão da inflamação sistêmica e agressividade do osteossarcoma

A PCR amplifica a resposta inflamatória ao ativar o sistema complemento e promover o recrutamento de células imunes para o microambiente tumoral. Esse estado inflamatório intensificado facilita a progressão tumoral por meio de vários mecanismos. Primeiro, a inflamação crônica pode aumentar a permeabilidade vascular e a angiogênese, ambas essenciais para que as células tumorais invadam os tecidos circundantes e entrem na corrente sanguínea. Segundo, as citocinas inflamatórias que aumentam a produção de PCR, como IL-6 e FNT-α, demonstraram promover a transição epitelial-mesenquimal (TEM), um processo pelo qual as células cancerígenas adquirem propriedades migratórias e invasivas necessárias para a metástase. 4 5

Além disso, a PCR tem sido implicada na supressão da imunidade antitumoral. Níveis altos de PCR podem inibir a atividade das células T citotóxicas e das células exterminadoras naturais ( *natural killer* , NK, em inglês), essenciais para o reconhecimento e a eliminação de células cancerígenas metastáticas. 21 Essa evasão imune permite que as células tumorais se disseminem com maior liberdade e causem lesões metastáticas em órgãos distantes.

### Velocidade de Hemossedimentação e Agressividade do Tumor

A VHS é outro marcador de inflamação sistêmica que reflete o grau de agregação de hemácias e é influenciado pela presença de proteínas de fase aguda, como fibrinogênio e PCR. A VHS elevada é indicativa de um processo inflamatório em andamento, e ela tem sido associada a desfechos adversos em diversos tipos de câncer. 7 22 Este estudo demonstra uma correlação significativa entre VHS alta e a presença de metástase, o que sugere que o aumento da inflamação sistêmica pode contribuir para o comportamento agressivo do osteossarcoma metastático.

A associação entre VHS elevada e metástase pode estar relacionada ao ambiente inflamatório crônico, que promove a progressão tumoral. Semelhante à PCR, a VHS elevada é provavelmente induzida por citocinas pró-inflamatórias, como IL-6 e FNT-α, que são conhecidas por desempenhar atuar na patogênese do osteossarcoma. Essas citocinas podem aumentar a capacidade invasiva das células tumorais, ao promover a quebra da matriz extracelular e facilitar a degradação das barreiras teciduais, o que permite sua migração e estabelecimento em locais de metástase. 23

### Razão Neutrófilo-Linfócito Como Indicador Prognóstico

A RNL é um marcador amplamente estudado na resposta inflamatória sistêmica, e representa o equilíbrio entre neutrófilos pró-tumorais e linfócitos antitumorais. A RNL elevada, como observada neste estudo, tem sido correlacionada a prognóstico ruim e aumento do potencial metastático de diversas neoplasias malignas, incluindo o osteossarcoma. A associação significativa entre a RNL elevada e a metástase no osteossarcoma destaca o papel dos neutrófilos na promoção do crescimento tumoral e dos linfócitos na defesa contra a progressão neoplásica. 24

Os neutrófilos contribuem para o processo metastático por meio de diversos mecanismos. Os neutrófilos associados a tumores (NATs) podem promover a angiogênese por meio da secreção de fator de crescimento endotelial vascular (FCEV), o que aumenta o suprimento sanguíneo para a neoplasia e facilita a disseminação metastática. Os NATs também liberam enzimas proteolíticas, como metaloproteinases de matriz (MPMs), que degradam a matriz extracelular e promovem a invasão das células tumorais. Além disso, os neutrófilos podem suprimir as respostas imunes adaptativas, inibindo a atividade de linfócitos T citotóxicos e células NK, o que facilita ainda mais a evasão imunológica e a metástase. 25

Por outro lado, os linfócitos, em especial os linfócitos T, são cruciais na imunidade antitumoral. O baixo número de linfócitos, refletido pela RNL elevada, indica menor vigilância imunológica e menor capacidade de controlar o crescimento tumoral e a metástase. Assim, a RNL elevada sugere um desequilíbrio entre as forças pró-tumorais e antitumorais, o que favorece a disseminação metastática. 25

### Lactato Desidrogenase e Metabolismo Tumoral

A LDH é uma enzima envolvida na glicólise anaeróbica, e seus níveis frequentemente estão elevados em células tumorais de rápida proliferação. 26 Altas concentrações de LDH têm sido associadas à biologia tumoral agressiva, resistência ao tratamento e desfechos ruins em casos de osteossarcoma. 2 Neste estudo, níveis elevados de LDH foram significativamente correlacionados à presença de metástase, o que ressalta seu possível papel como marcador de agressividade tumoral e potencial metastático.

A relação entre LDH e metástase pode ser compreendida no contexto do efeito de Warburg, uma adaptação metabólica em que as células cancerígenas utilizam preferencialmente glicólise para a produção de energia, mesmo na presença de oxigênio. Esse favorecimento à glicólise aumenta a produção de lactato, que acidifica o microambiente tumoral e promove a invasão neoplásica e a metástase. Níveis elevados de LDH refletem essa alteração metabólica e geralmente são associados ao aumento da proliferação de células tumorais e da capacidade metastática. 27

Além disso, o próprio lactato pode atuar como uma molécula sinalizadora no microambiente tumoral, ao promover angiogênese, evasão imune e remodelação tissular, processos que facilitam a metástase. Altas concentrações de LDH também refletem as condições hipóxicas dentro do tumor, que são conhecidas por impulsionar a seleção de clones celulares metastáticos mais agressivos. 28

Apesar da associação estatisticamente significativa entre os marcadores inflamatórios e metástase aqui demonstrada, este estudo tem várias limitações. O delineamento retrospectivo depende de prontuários médicos preexistentes, que podem ter qualidade e completude variáveis. Essa dependência pode introduzir viés de seleção devido à inclusão apenas de pacientes que atenderam aos critérios especificados e tinham dados completos à disposição, com a possível exclusão de casos que poderiam influenciar os resultados. Além disso, a natureza retrospectiva limita o controle dos fatores de confusão, como variações nas técnicas diagnósticas ou protocolos de tratamento, que podem influenciar os resultados do estudo. Essas limitações podem afetar a generalização dos achados para populações mais amplas, o que ressalta a necessidade de estudos prospectivos para a validação desses resultados e esclarecimento dos mecanismos etiológicos.

## Conclusão

Este estudo demonstra que VHS e RNL altas e níveis elevados de PCR, neutrófilos e LDH estão significativamente associados à metástase em pacientes com osteossarcoma, o que destaca o valor prognóstico dos marcadores inflamatórios sistêmicos e seu possível papel na orientação da estratificação de risco e estratégias terapêuticas personalizadas.
